# Randomized, double-blind, placebo-controlled trial of arimoclomol in rapidly progressive *SOD1* ALS

**DOI:** 10.1212/WNL.0000000000004960

**Published:** 2018-02-13

**Authors:** Michael Benatar, Joanne Wuu, Peter M. Andersen, Nazem Atassi, William David, Merit Cudkowicz, David Schoenfeld

**Affiliations:** From the Department of Neurology (M.B., J.W.), University of Miami, FL; Department of Pharmacology and Clinical Neuroscience (P.M.A.), Umeå University, Sweden; Department of Neurology (N.A., W.D., M.C.), Massachusetts General Hospital (D.S.), Harvard Medical School; and Department of Biostatistics (D.S.), Harvard Chan School of Public Health, Boston, MA.

## Abstract

**Objective:**

To examine the safety and tolerability as well as the preliminary efficacy of arimoclomol, a heat shock protein co-inducer that promotes nascent protein folding, in patients with rapidly progressive *SOD1* amyotrophic lateral sclerosis (ALS).

**Methods:**

This was a double-blind, placebo-controlled trial in which patients with rapidly progressive *SOD1*-mutant ALS were randomized 1:1 to receive arimoclomol 200 mg tid or matching placebo for up to 12 months. Study procedures were performed using a mix of in-person and remote assessments. Primary outcome was safety and tolerability. Secondary outcome was efficacy, with survival as the principal measure. Additional efficacy measures were the rates of decline of the Revised ALS Functional Rating Scale (ALSFRS-R) and percent predicted forced expiratory volume in 6 seconds (FEV6), and the Combined Assessment of Function and Survival (CAFS).

**Results:**

Thirty-eight participants were randomized. Thirty-six (19 placebo, 17 arimoclomol) were included in the prespecified intent-to-treat analysis. Apart from respiratory function, groups were generally well-balanced at baseline. Adverse events occurred infrequently, and were usually mild and deemed unlikely or not related to study drug. Adjusting for riluzole and baseline ALSFRS-R, survival favored arimoclomol with a hazard ratio of 0.77 (95% confidence interval [CI] 0.32–1.80). ALSFRS-R and FEV6 declined more slowly in the arimoclomol group, with treatment differences of 0.5 point/month (95% CI −0.63 to 1.63) and 1.24 percent predicted/month (95% CI −2.77 to 5.25), respectively, and the CAFS similarly favored arimoclomol.

**Conclusions:**

This study provides Class II evidence that arimoclomol is safe and well-tolerated at a dosage of 200 mg tid for up to 12 months. Although not powered for therapeutic effect, the consistency of results across the range of prespecified efficacy outcome measures suggests a possible therapeutic benefit of arimoclomol.

**Clinicaltrials.gov identifier:**

NCT00706147.

**Classification of evidence:**

This study provides Class II evidence that arimoclomol is safe and well-tolerated at a dosage of 200 mg tid for up to 12 months. The study lacked the precision to conclude, or to exclude, an important therapeutic benefit of arimoclomol.

Amyotrophic lateral sclerosis (ALS) is characterized by the formation of cytosolic aggregates that contain specific misfolded proteins in selected neuronal and glia cells.^1^ There is mounting evidence that these aggregates play a pathogenic role in disease initiation and propagation.^2,3^ Mutations in the gene encoding CuZn-superoxide dismutase (SOD1), the second most common identified cause of ALS, make the SOD1 protein more prone to aggregation, resulting in the deposition of cellular inclusions that contain misfolded SOD1 aggregates.^4^ With the goal of targeting the formation of such pathogenic aggregates, we selected arimoclomol (CytRx, Los Angeles, CA; and Orphazyme, Copenhagen, Denmark) for evaluation in patients with rapidly progressive ALS caused by *SOD1* mutations. Arimoclomol is a co-inducer of the heat shock protein (HSP) response^5–7^ and promotes natural folding of nascent proteins and refolding of misfolded proteins.^8^ Our choice of arimoclomol was further supported by evidence of meaningful therapeutic effect in methodologically rigorous studies in the G93A *SOD1* mouse^7,9^; its good safety record (up to 300 mg/day) for a short period of time (12 weeks) in patients with ALS; and evidence of good penetration across the blood–brain barrier.^10^

Since ALS is an etiologically, genetically, and phenotypically heterogeneous syndrome,^11,12^ we elected to focus exclusively on patients with ALS with a subset of *SOD1* mutations that result in unstable SOD1 proteins^13^ and are associated with a uniformly rapid rate of disease progression.^14^ We were cognizant that rapid disease progression would yield both advantages (e.g., large degree of measurable functional decline) and disadvantages (e.g., a highly aggressive form of disease might be most impervious to therapeutic efforts). We also recognized that the rapid accumulation of physical disability could limit participants' ability to travel for study visits, and that recruitment would be challenging for this ultrarare population. For these reasons, this study was originally proposed as an adaptive design, seamless, phase II/III, randomized, double-blind, placebo-controlled trial, with remote assessment of outcomes. The initial goals were to show safety/tolerability (phase II) and then efficacy (phase III). Slow recruitment, however, necessitated an administrative decision to close the trial after completion of phase II. Here we report the safety and efficacy data from the completed phase II component of this trial.

## Methods

### Standard protocol approvals, registrations, and patient consents

Institutional review board (IRB) approval was obtained at each study center, and all participants provided written informed consent. The trial was registered on clinicaltrials.gov (NCT00706147).

### Study design and participants

This randomized, double-blind, placebo-controlled trial was conducted at 2 sites and 3 academic medical centers in the United States (primary site: Emory University [until February 2011] then University of Miami [from March 2011]; second site: Massachusetts General Hospital [MGH]). An independent medical monitor completed regular review of laboratory reports and adverse events (AEs), as well as real-time review of serious AEs (SAEs). Eligibility criteria (table e-1, links.lww.com/WNL/A135) aimed to enroll, from across the United States and Canada, a population relatively early in the course of ALS caused by *SOD1* mutations associated with rapidly progressive disease.

### Randomization and blinding

Randomization (1:1 to arimoclomol or matching placebo) was stratified by riluzole use and in block size of 2 within each stratum, and implemented using a central web-based electronic data capture (EDC) system managed by the Neurologic Clinical Research Institute Data Management Center at MGH. The randomization schedule, generated by a study statistician (J.W.), was provided to each research pharmacy. At randomization, the EDC assigned a participant identification number, which the site coordinator submitted to the research pharmacy for drug dispensing. Encapsulated placebo was matched in color and appearance to active drug. Except for the research pharmacists, pharmacy monitors, and study statisticians, all other personnel and study participants were blinded to treatment assignments.

### Procedures

Arimoclomol and matching placebo were prepared and packaged by the CytRx Corporation (Los Angeles, CA). The research pharmacy of the primary site labeled and distributed investigational product to the second study site. When the first participants were randomized (February 2009), the highest dose permitted under the active Investigational New Drug (IND) was 100 mg tid. In May 2010, after enrollment of the first 16 participants (of whom 8 were on arimoclomol, including 1 who was subsequently excluded per protocol), the Food and Drug Administration approved the use of 200 mg tid. Dosage was increased to this level for all active participants (which for the arimoclomol group included 3 initially started at 100 mg tid), and all newly enrolled participants received 200 mg tid after IRB approval of the revised protocol. All data were analyzed following the intent-to-treat principle.

Knowing that participants, to be recruited across a broad geographic area, would accumulate physical disability quickly, we anticipated the challenges that participants would face in order to travel to a study site. By design, therefore, assessments were performed in person only at baseline and month 2, with remote assessments planned for all other visits. Neurologic examination, motor unit number estimation, and slow vital capacity (SVC) could only be performed in person. Other assessments, conducted at all visits (in person and remotely), included vital signs; blood and urine for safety laboratories; Revised ALS Functional Rating Scale (ALSFRS-R), which has been validated for telephone administration^15^; and the forced expiratory volume in 6 seconds (FEV6).^16^ In addition, AEs, concomitant medications, study drug dose management, use of ventilatory support, and key study events were recorded at all visits.

Operationalization of remote assessments posed logistical challenges that required the introduction of 2 innovative features: (1) the collection of vital signs and blood/urine for safety laboratories in participants' homes: this was accomplished using the services of a mobile medical provider, Examination Management Services, Inc.; and (2) the use of FEV6 as the principal measure of respiratory function: the key advantage of FEV6 over SVC—and indeed, a necessity for this study—is that FEV6 may be self-administered by the participant using a low-cost portable device (Piko-6; nSpire Health, Inc., Longmont, CO). The device digitally displayed the FEV6 value, which the participant then reported to the study coordinator. Our use of the FEV6 in this way allowed for remote assessment of respiratory function, which we would otherwise not have been able to collect in this trial. Reproducibility and normative data for FEV6 are established.^17^

### Outcomes

The primary outcome measure was originally designated as the ALSFRS-R rate of decline over 12 months, with survival (defined as permanent assisted ventilation [PAV] and tracheostomy-free survival) and FEV6 rate of decline as secondary measures. Given the higher than expected mortality observed in the combined groups in the blinded data, the principal efficacy measure was changed to survival in February 2014 with approval of the Data Safety Monitoring Board (DSMB). Moreover, following the administrative decision to close the trial early, safety became the designated primary outcome, with efficacy secondary. These revisions to the protocol and statistical analysis plan were submitted under IND 101,942.

The primary endpoint of safety and tolerability was based on the frequency of AEs, abnormal vital signs, and abnormal laboratory studies above or below predefined alert levels. AEs and SAEs were categorized according to the Common Terminology Criteria for Adverse Events (CTCAE) and rated for severity and relatedness to study drug. In summarizing AEs: (1) if a participant experienced multiple occurrences of the same event, only the occurrence with the worst severity (or highest degree of relatedness to study drug) was counted; (2) CTCAE events were further classified into subgroups (AE type); e.g., pneumonia and bronchitis were both classified as upper/lower respiratory infection.

For efficacy, the principal outcome measure was PAV- and tracheostomy-free survival time, calculated from baseline to PAV (defined as first of 7 consecutive days when PAV was used >22 hours/d) or tracheostomy, date of death (if no PAV or tracheostomy), or date of last available follow-up/study contact (if still PAV- and tracheostomy-free by then). Participants not reaching survival endpoints were censored. Secondary efficacy measures included ALSFRS-R rate of decline (points/month), FEV6 rate of decline (% predicted/month), and joint rank scores of the Combined Assessment of Function and Survival (CAFS), which considers both ALSFRS-R rate of decline and survival.

### Sample size and statistical analyses

With insufficient published data to establish a fixed sample size, we initially proposed a seamless adaptive design using methods described by Fisher,^18^ with phase III sample size to be re-estimated at the end of phase II, once the first 30 participants had completed at least 6 months of follow-up. The phase II target sample size of 30 was based on our consideration of the acceptable 6-month treatment failure rate in the arimoclomol group, with treatment failure defined as failure to remain on the originally assigned treatment and dose due to occurrence of an AE. Specifically, in the 15 arimoclomol participants we would estimate the 80% confidence interval (CI) of their 6-month treatment failure rate, which was expected to be <40% based on the preliminary estimate of 13.6% in a prior arimoclomol trial.^10^ The acceptance of such a high rate of treatment failure (and wide CI) was justified by the inexorably progressive nature of the disease and the absence of any known effective therapy. We prespecified that we would consider arimoclomol tolerable if the upper bound of this CI was <40%. At the end of phase II, we were to utilize all available data (up to 12 months of follow-up) to estimate the number of additional participants needed in phase III in order to detect, with 80% power and a 2-sided *p* = 0.05 significance level, a treatment difference of 30% based on ALSFRS-R rate of decline, which was the difference deemed clinically meaningful and had been used in previous trials.^19^ The trial, however, was closed before sample size re-estimation.

All 36 eligible participants who completed at least one follow-up visit were included in the intent-to-treat analysis. The only predefined subgroup was the A4V *SOD1* mutation carriers, whom we expected to be the largest genetic subgroup and to exhibit a uniformly aggressive disease course. An independent DSMB performed periodic review of cumulative safety data and made recommendations to the principal investigator regarding trial continuation.

PAV- and tracheostomy-free survival was first summarized by Kaplan-Meier survival estimates and compared between treatment groups by Wilcoxon and log-rank tests, then analyzed using a proportional hazards model with riluzole use and baseline ALSFRS-R as prespecified covariates. ALSFRS-R and FEV6 rates of decline were compared between groups by mixed model analysis with a random intercept and slope, and the outcome measure at each visit as dependent variable. The independent variables were time and time–treatment interaction, with the test of treatment effect based on the time–treatment interaction. In secondary analyses, a quadratic term for time was included, as suggested by the finding of another ALS trial.^20^ In addition, 2 analyses that combine survival and ALSFRS-R rate of decline were performed. The CAFS joint rank scores^21^ were compiled for each participant and compared between groups by 2-sample *t* test. Treatment effect on the ALSFRS-R rate of decline, as well as any treatment effect on survival that is mediated through the ALSFRS-R, were estimated by the Vonesh shared parameter model.^22^ Numerical time, based on actual number of days between baseline and each follow-up visit, was used in the survival, mixed model, and Vonesh analyses. For CAFS, survival times and ALSFRS-R rates of decline were obtained using numerical time, but the participant-to-participant comparisons made at nominal time points. Baseline covariate adjustment for potential imbalance between groups was considered. All analyses were performed using SAS 9.3.

### Role of the funding source

None of the funding sources played any role in the design, analysis, or interpretation of data. The trial was conducted under the auspice of an investigator-sponsored IND, referencing the parent IND initially held by the CytRx Corporation and, after May 2011, by Orphazyme ApS (Copenhagen, Denmark). Neither CytRx nor Orphazyme played any role in trial design/implementation or in data analysis/publication.

## Results

### Participants

**Figure 1:**
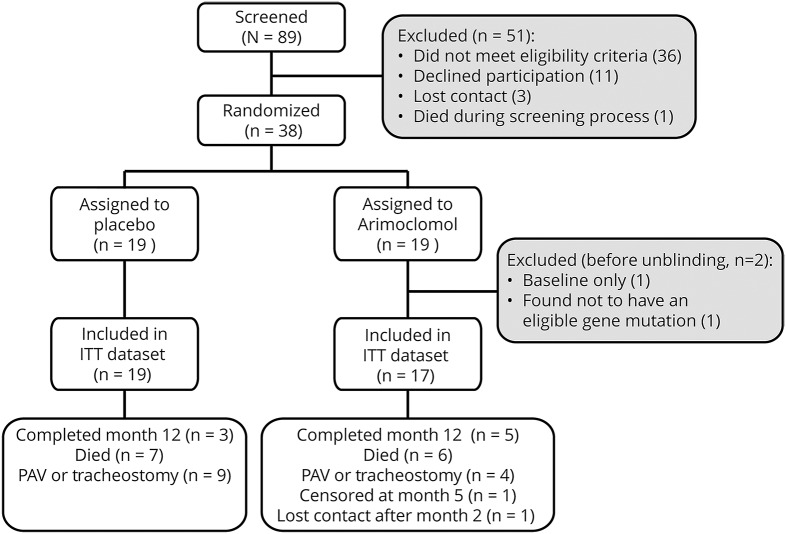
Consort diagram There were 2 patients in the arimoclomol group who were excluded from the intent-to-treat (ITT) analysis based on prespecified criteria: 1 patient died before completion of the month 1 visit; the other was found, after randomization, not to have a mutation in the *SOD1* gene. The 1 patient in the arimoclomol group who was censored at month 5 had stopped drug at month 2 because of a skin rash thought probably related to study drug, and was then followed until month 5, when the participant enrolled in another clinical trial. PAV = permanent assisted ventilation.

Enrollment was open between December 2008 and June 2014. The administrative decision to close the trial early was made by the steering committee, with input from the DSMB, while blinded to treatment group and study results. A total of 89 patients were screened, and 38 randomized (19/group). The absence of an *SOD1* mutation (n = 18) and the presence of an ineligible *SOD1* mutation (n = 8) were the most common reasons for ineligibility. The 38 randomized were recruited at Emory (n = 15), University of Miami (n = 13), and MGH (n = 10). Per protocol, 2 participants were excluded from analysis; one did not have an eligible *SOD1* mutation and the other completed no follow-up visits after baseline (figure 1). Exclusions were made before unblinding, and both later revealed to be in the arimoclomol group. The analysis dataset thereby comprised 19 (53%) participants on placebo and 17 (47%) participants on arimoclomol. Groups were balanced at baseline except that FEV6 and SVC were higher in the arimoclomol group, while body mass index was higher in the placebo group (table 1). Baseline SVC and FEV6 were strongly correlated (*r* = 0.89, *p* < 0.0001).

**Table 1:**
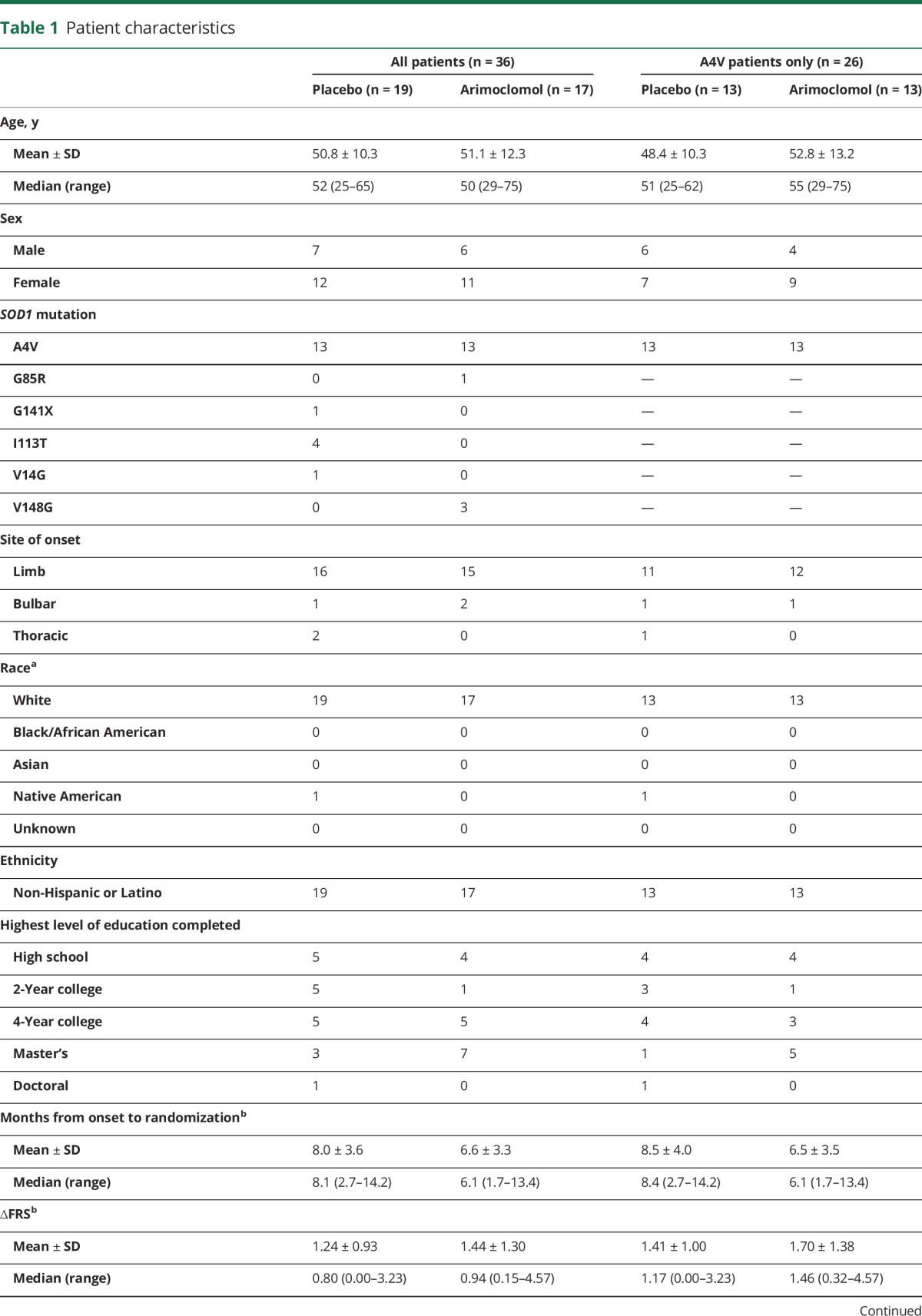

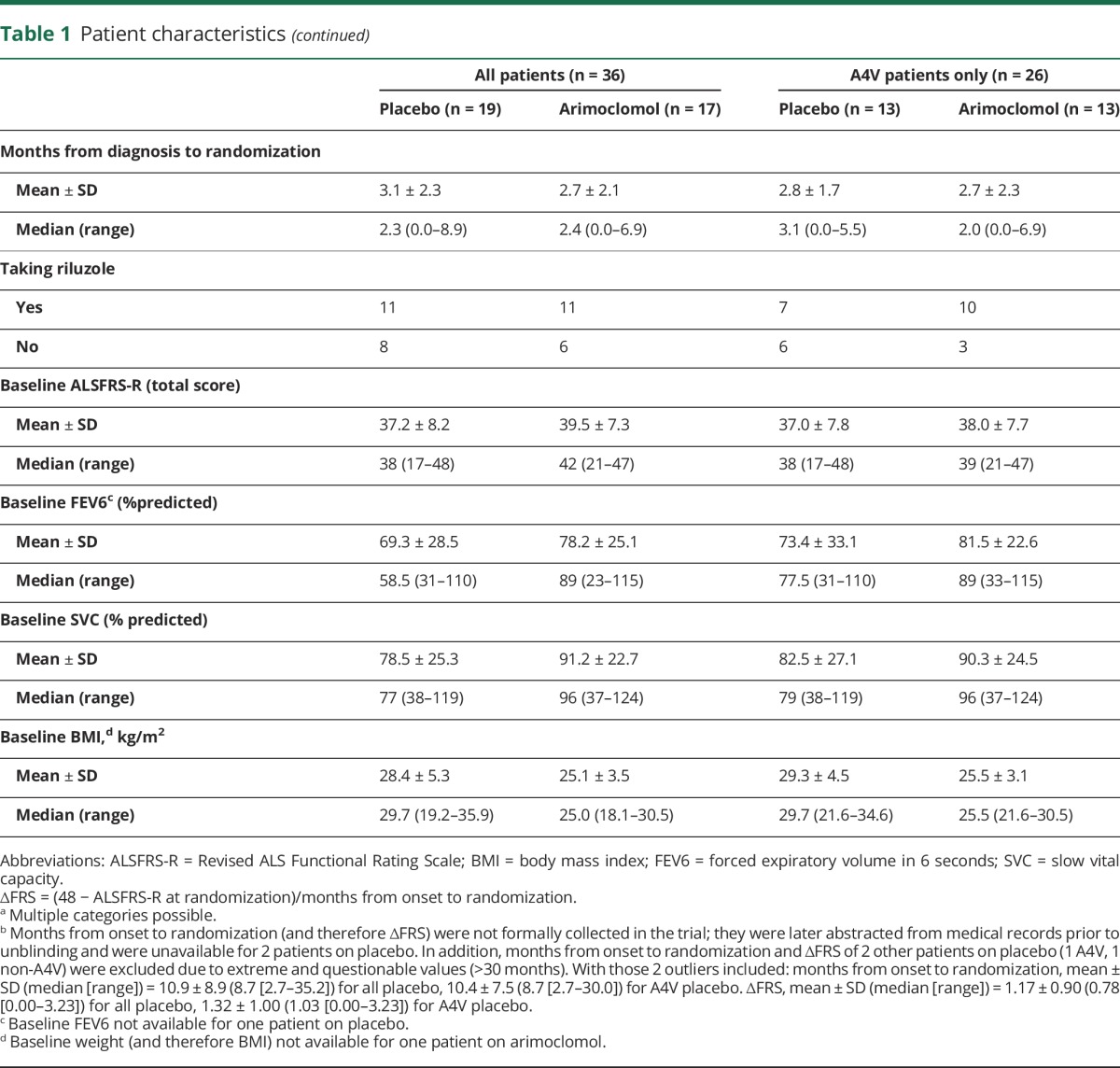
Patient characteristics

### Safety

AEs occurring in ≥10% (n ≥ 4) of participants are summarized in table 2. These were generally mild, occurred with similar frequency in the 2 arms, and were largely considered unrelated to study drug. Twenty-two SAEs were reported (15 in the placebo and 7 in the arimoclomol group), none of which were considered related to study drug. Abnormal vital signs or laboratory values were infrequent and occurred with comparable frequency in the 2 groups (table e-2, links.lww.com/WNL/A135). A single participant stopped arimoclomol because of a skin rash (probably related to study drug).

**Table 2:**
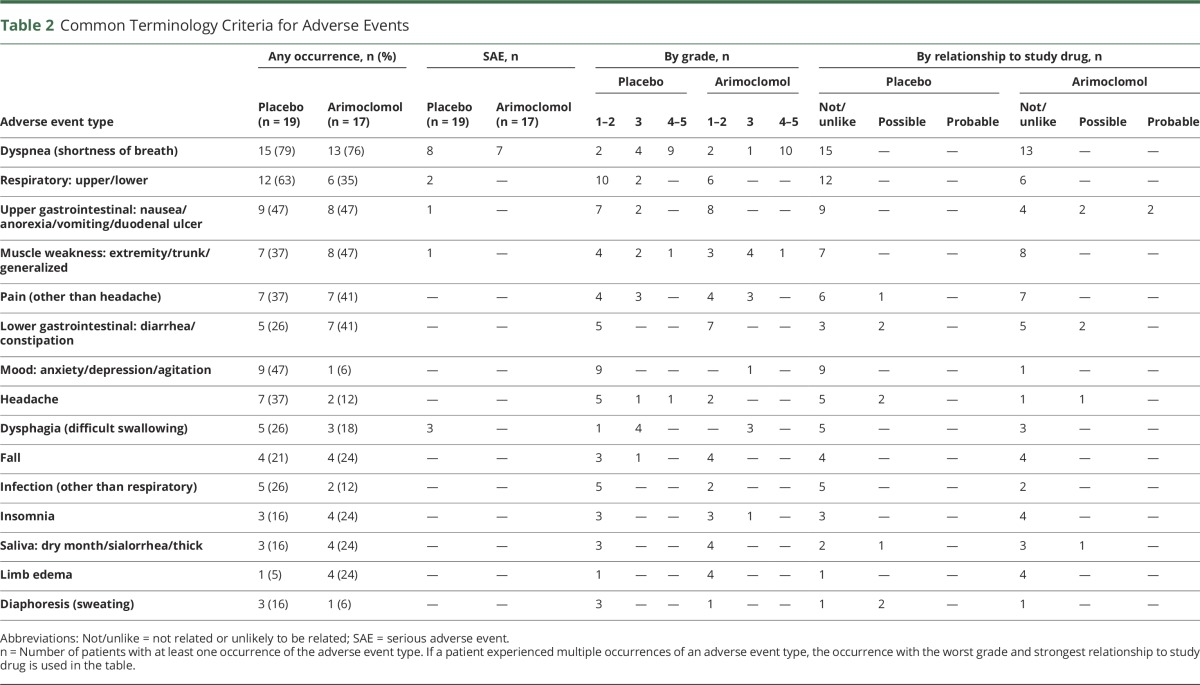
Common Terminology Criteria for Adverse Events

### Efficacy

**Figure 2:**
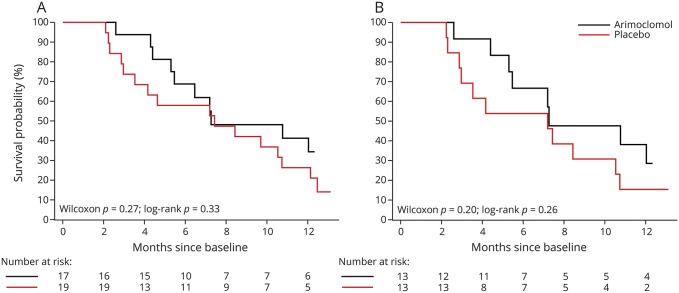
Permanent assisted ventilation (PAV)– and tracheostomy-free survival (A) All arimoclomol- and placebo-treated participants. At 12 months, 34% of arimoclomol-treated and <21% of placebo-treated participants were alive (and without PAV or tracheostomy). (B) The subgroup of A4V participants. At 12 months, 29% of arimoclomol-treated and 15% of placebo-treated participants were alive (and without PAV or tracheostomy).

While none of the efficacy analyses yielded statistically significant results, all point estimates favored arimoclomol but with CIs spanning unity. Kaplan-Meier plot shows a separation in survival curves, though the curves slightly crossed around 7.5 months (Wilcoxon test *p* = 0.27, log-rank test *p* = 0.33) (figure 2A). Similar results are observed in the A4V subgroup (13/group) (figure 2B). Survival estimates from the Cox proportional hazards model also favored arimoclomol compared to control, with an unadjusted hazard ratio (HR) of 0.67 (95% CI 0.29–1.48, *p* = 0.33); adjusting for baseline ALSFRS-R and riluzole, HR 0.77 (95% CI 0.32–1.80, *p* = 0.55). Adjustment for the baseline imbalance in respiratory function did not affect results (table e-3, links.lww.com/WNL/A135).

Among placebo-treated participants, ALSFRS-R declined by an average (±SE) of 3.0 ± 0.4 points/month, compared to 2.5 ± 0.4 points/month in the arimoclomol-treated group, a treatment difference of 0.5 points/month (95% CI −0.63 to 1.63, *p* = 0.37). In the A4V subgroup, ALSFRS-R declined even faster, by an average of 3.6 ± 0.5 points/month in the placebo group but only 2.6 ± 0.5 points/month in the arimoclomol group, a treatment difference of 0.98 points/month (95% CI −0.28 to 2.24, *p* = 0.12). Notably, the magnitude of this difference is comparable to the average ALSFRS-R rate of decline in the untreated general ALS population (1.02 ± 2.3 points/month).^23^ Moreover, the treatment difference of 0.5–1.0 points/month in ALSFRS-R rate of decline, though not statistically significant, translates into a clinically meaningful difference of 6–12 points over a 1-year period. Similar results were observed for the FEV6% predicted rate of decline (table 3). Moreover, while adjustment for baseline ALSFRS-R or respiratory function variably increased or decreased the magnitude of treatment effect, overall results were unchanged (table e-3, links.lww.com/WNL/A135).

**Table 3:**
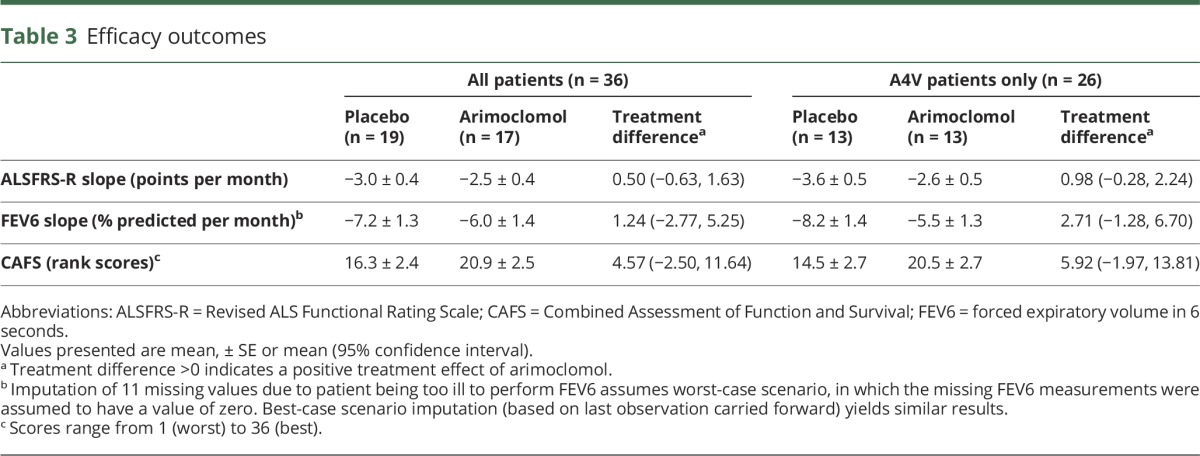
Efficacy outcomes

For the CAFS, which weights mortality as the more clinically important outcome and whose rank scores range from 1 (worst) to 36 (best), the average (±SE) score in the arimoclomol group was 20.9 ± 2.5 compared to 16.3 ± 2.4 in the placebo group, with a potential treatment benefit of 4.57 points (95% CI −2.50 to 11.64) (table 3). Moreover, in participant-to-participant comparison between the 2 groups, the arimoclomol group had a clinically significant win ratio of 1.69.^24^ The Vonesh model, on the other hand, yielded a treatment difference of 0.77 ± 0.54 points/month (95% CI −0.33 to 1.86, *p* = 0.16) in ALSFRS-R rate of decline, whereas the direct effect of treatment on survival was not significant (*p* = 0.62). This would indicate that the possible survival benefit of arimoclomol was mediated through a slowed decline of the ALSFRS-R rather than through an independent effect on survival.

## Discussion

Arimoclomol was safe and well-tolerated at a dose of 200 mg tid. While not powered to detect a statistically significant therapeutic effect, the consistency of results across all prespecified efficacy measures suggests a possible therapeutic benefit of arimoclomol in the overall study population and especially in the A4V subgroup.

We do not interpret these results as showing efficacy, but are encouraged by the strength and consistency of these preliminary findings. Moreover, this trial is significant in 3 other respects. (1) It represents the first ALS trial initiated in a genetically and phenotypically homogeneous population. Although logistically challenging, this approach is both feasible and highly relevant to future trials, as we increasingly recognize the importance of targeting drugs with particular mechanisms of action to patient subpopulations most likely to benefit. (2) This trial pioneered an approach in which safety and efficacy assessments relied heavily on remote assessments. This key design feature helped to mitigate the physical burden of travel to a study center, which can be a major barrier to trial participation. We have also introduced FEV6 to the ALS field and demonstrated its usefulness in reliably quantifying respiratory function; FEV6 may be a useful alternative to vital capacity when the latter cannot readily be obtained. (3) This trial provides the first prospectively acquired natural history data that inform survival in the mutant *SOD1* population. Among the placebo-treated patients, median survival from diagnosis was 11.1 months (95% CI 6.5–13.5) for all *SOD1* mutations, and 9.3 months (95% CI 6.1–12.5) in the A4V subgroup. Similarly, median survival from baseline was 7.4 months (95% CI 3.0–10.7) and 7.2 months (95% CI 2.9–10.5), respectively. These estimates are of particular relevance to future therapeutic studies. Moreover, median disease duration (time from symptom onset to survival endpoint) was 17.5 months (95% CI 11.6–18.4) among all placebo-treated patients, and 13.0 months (95% CI 6.3–21.3) in the A4V population; these data lend weight to estimates of disease duration based on retrospectively collected data.^14,25^

An important limitation of this trial is the small sample size, which was a function of the ultrarare population targeted for enrollment. We estimate that at most 320 patients across the entire United States could have been considered for the trial over its planned 5-year duration, and this was assuming that all patients would be willing to participate, and without accounting for other eligibility criteria. Moreover, while this study was ongoing, 2 other trials targeting the mutant *SOD1* population were also recruiting.^26,27^ Although we successfully enrolled >10% of the theoretically eligible population, it was insufficient to permit completion of the planned phase III component of the trial.

The absence of wet biomarker data is another limitation. This reflects the logistical complexity and cost that would have been required to collect, process, and store biological samples from patients who were largely evaluated remotely. Growing interest in developing methods or devices for remote collection of biomarker data might help to mitigate such challenges in future trials.

These data support further development of arimoclomol as a potential therapeutic for ALS. Critical to the design of the next study will be the incorporation of potential pharmacodynamic biomarkers, including those that might show target engagement (e.g., upregulation of HSP). Moreover, it will be essential to explore the use of a higher dose of arimoclomol and to broaden the eligibility criteria to include a more diverse population of patients with ALS, since arimoclomol's mechanism of action is likely to be relevant to all forms of ALS, in which aberrant proteostasis plays an essential role in disease pathophysiology.

## Glossary

AE: adverse event
ALS: amyotrophic lateral sclerosis
ALSFRS-R: Revised ALS Functional Rating Scale
CAFS: Combined Assessment of Function and Survival
CI: confidence interval
CTCAE: Common Terminology Criteria for Adverse Events
DSMB: Data Safety Monitoring Board
EDC: electronic data capture
FEV6: forced expiratory volume in 6 seconds
HR: hazard ratio
HSP: heat shock protein
IND: Investigational New Drug
IRB: institutional review board
MGH: Massachusetts General Hospital
PAV: permanent assisted ventilation
SAE: serious adverse event
SOD1: CuZn-superoxide dismutase
SVC: slow vital capacity
